# Evolutionary history of adenomas to colorectal cancer in FAP families

**DOI:** 10.3389/fgene.2024.1391851

**Published:** 2024-07-03

**Authors:** Cuiping Yang, Enfei Xiang, Ping Chen, Xuqian Fang

**Affiliations:** ^1^ Department of Gastroenterology, Ruijin Hospital, Shanghai Jiao Tong University School of Medicine, Shanghai, China; ^2^ Shanghai Institute of Digestive Surgery, Ruijin Hospital, Shanghai Jiao Tong University School of Medicine, Shanghai, China; ^3^ Department of Pathology, Ruijin Hospital, Shanghai Jiao Tong University School of Medicine, Shanghai, China

**Keywords:** familial adenomatous polyposis (FAP), adenoma-carcinoma transition, clonal evolution, APC, chromosomal instability, DNA damage repair genes

## Abstract

**Objective:**

Familial adenomatous polyposis (FAP) is a genetic syndrome characterized by multiple polyps at various evolutionary stages, which, if left untreated, inevitably progress to colorectal cancer (CRC). In this study, we present a comprehensive analysis of the evolutionary history of FAP-CRC from precancerous adenoma to carcinoma.

**Design:**

Tissues were collected from gastrointestinal endoscopy or surgical resection. Exome sequencing was performed on multiple regions of adenocarcinoma (*n* = 8), villous adenoma (*n* = 10), tubular adenoma (*n* = 9) and blood samples were obtained from 9 patients belonging to 7 Chinese FAP families. Phylogenetic trees were reconstructed, and evolutionary analysis was conducted to reveal the temporal sequence of events leading to CRC.

**Results:**

Inherited germline mutation sites in *APC* gene were identified in FAP01 (p.S1281*, COSM19212), FAP03 (p.S384Tfs*19), FAP04 (p.E1538*, COSM6041693), FAP05 (p.Q1062*, COSM3696862), and FAP07-FAP09 (p.V677Sfs*3). Notably, p.V677Sfs*3 mutation was recognized as a novel germline mutation in *APC*, supported by evidence of genotype-phenotype correlation in pedigree analysis. Adenomas exhibited lower mutational rates than FAP-CRC and displayed recurrent alterations in well-known chromosomal instability (CIN) genes (*APC, RAS*, *SMAD4* and *TP53*) and DNA damage repair genes (*SUZ12*, *KMT2C, BCLAF1*, *RUNX1*, and *ARID1B*), suggesting the presence of genomic instability. Furthermore, a progressive increase in the HRD score (a measure of “genomic scars”) was observed from tubular adenomas to villous adenomas and ultimately to carcinomas. *TP53* emerged as the primary driver gene for adenoma-carcinoma transition, with driver mutations consistently appearing simultaneously rather than sequentially acquired from adenomas to carcinomas. Clonal evolution demonstrated that liver metastases can originate from the same cancer-primed cell present in a primary cancerous lesion.

**Conclusion:**

We identified a novel pathogenic variant in *APC,* namely, p.V677Sfs*3. The process of carcinogenesis in FAP-CRC supports the classical cancerization model, where an initial *APC* mutation leads to the activation of the WNT signaling pathway and CIN. Subsequently, additional mutations occur in other putative CIN genes (e.g., DNA repair, chromatin remodeling), ultimately leading to the development of microsatellite stable (MSS) tumors. Our study provides a comprehensive understanding of the genomic landscapes that underlie the transition from adenoma to carcinoma.

## Introduction

Familial adenomatous polyposis (FAP) is an autosomal dominant syndrome primarily caused by inherited mutations in the APC gene. Patients with FAP typically develop hundreds to thousands of adenomas in the colon and rectum, which if left untreated, will inevitably progress to adenocarcinomas (Bisgaard et al., 1994). The *APC* gene functions as a tumor suppressor, regulating the WNT signaling pathway and maintaining genomic stability. Loss of *APC* function, resulting from mutations or deletion, leads to increased β-catenin levels and persistent activation of the WNT signaling pathway, which can trigger genomic instability and promote tumor development (Kinzler et al., 1991; Morin et al., 1997). While approximately 85% of sporadic colorectal cancers also exhibit somatic *APC* mutation, FAP presents with multiple polyps in different stages of carcinogenesis, make it an ideal natural model for tracing the progression of colorectal carcinogenesis.

High throughput sequencing approaches, such as whole-exome sequencing (WES), whole-genome sequencing (WGS) or Single-Cell RNA-seq (Cancer Genome Atlas, 2012; Borras et al., 2016; Li et al., 2020), have been employed to investigate the differences between adenomas and carcinomas, aiming to identify crucial events in the progression of colorectal cancer. However, the variations among lesions obtained from a large cohort may be confounded by the inter-individual factors, such as genetic background, dietary habits and intestinal flora. In this study, we adopted a unique approach by collecting adenomas at different stages and carcinomas from the same patient. Specifically, we continuously collected lesions at various stages of the cancer progression course from three FAP patients within the same family, over a period of 5 years. Every lesion collected was spatially or time independent. The genomic landscapes and clonal architecture of lesions at different evolutionary stages were comprehensively investigated.

## Materials and methods

### Sample collection

Tissues were collected from gastrointestinal endoscopy during annual physical examinations or from curative resections of colorectal cancer. The collected lesions underwent evaluation through haematoxylin and eosin (H&E) staining as well as immunohistochemical staining for pathological analysis. FFPE or fresh tissues, and blood samples were subjected to whole-exome sequencing (WES). WES sequencing was performed on multiple regions of adenocarcinoma (*n* = 8), villous adenoma (*n* = 10), tubular adenoma (*n* = 9) and blood samples obtained from 9 patients belonging to 7 Chinese FAP families.

### Whole-exome sequencing

Whole-exome sequencing was conducted utilizing the Agilent whole-exome capture kit (SureSelectXT Human All Exon 50 Mb), as described previously (Coffey et al., 2011). Briefly, total DNA was extracted from the collected specimens using a standard DNA extraction protocol. The extracted genomic DNA was sonicated to generate fragments of 150–200 bp. Subsequently, the fragmented DNA underwent processing and preparation for sequencing using a whole-exome library preparation kit, following the manufacturer’s guidelines. Multiple libraries, each with distinct barcode adaptors, were pooled together for the whole-exome sequence capture step.

### Sanger sequencing

To verify the APC germline mutation sites, Sanger sequencing was employed. The primers utilized for Sanger sequencing are summarized in online Supplementary Table S1.

### Data analysis

#### Calculating tumor mutation burden (TMB) based on whole-exome sequencing (WES) data

Variant Calling and Filtering: Perform variant calling and filtering on the tumor and matched normal samples to identify high-quality somatic mutations. Exome Size Calculation: Calculate the size of the exome based on the sequencing coverage and the exome capture kit used. This information is usually provided by the sequencing center or can be estimated from the sequencing data itself. Mutation Count: Count the number of somatic mutations identified within the exome. Make sure to exclude any germline mutations and filter out variants that are likely to be artifacts. Calculate TMB: Calculate TMB by dividing the number of somatic mutations by the size of the exome (in megabases). This yields the TMB score in mutations per megabase. Adjust for Sequencing Depth: Adjust the TMB score to account for differences in coverage. This can be achieved by weighting the mutations based on the sequencing depth at each genomic position in the exome.

#### Genomic scar scores and HRDetect

The calculation of the genomics scar scores [LOH (Abkevich et al., 2012); large-scale transitions (LST; Popova et al., 2012); and number of telomeric allelic imbalances (ntAI; Birkbak et al., 2012) were determined using the scarHRD R package (Sztupinszki et al., 2018). Due to the lack of a colon cancer-specific HRDetect model, instead, the scores of the WES samples were calculated by using the weights of a whole exome specific model.

### Clonal evolution

Heterozygous mutations were first clustered on the basis of their variant allele fraction using sciClone (Miller et al., 2014) to identify the founding clones and subclones that were subsequently analyzed using ClonEvol (Dang et al., 2017) to infer clonal evolution models.

#### Statistical analysis

The descriptive data were expressed as the mean value (standard deviation), median and range (25 and 75th percentiles), or frequencies (%). Tumor mutation burden (TMB) and homologous recombination deficiency (HRD) scores across three groups were compared utilizing one-way analysis of variance (ANOVA). The differences between pairs of groups were assessed using the independent Student’s *t*-test. All statistical analyses were conducted with GraphPad Prism software, version 8.0. The probability values reported are two-tailed, and statistical significance was determined at a threshold of *p* < .05.

## Results

### Overview of the cohort

**FIGURE 1:**
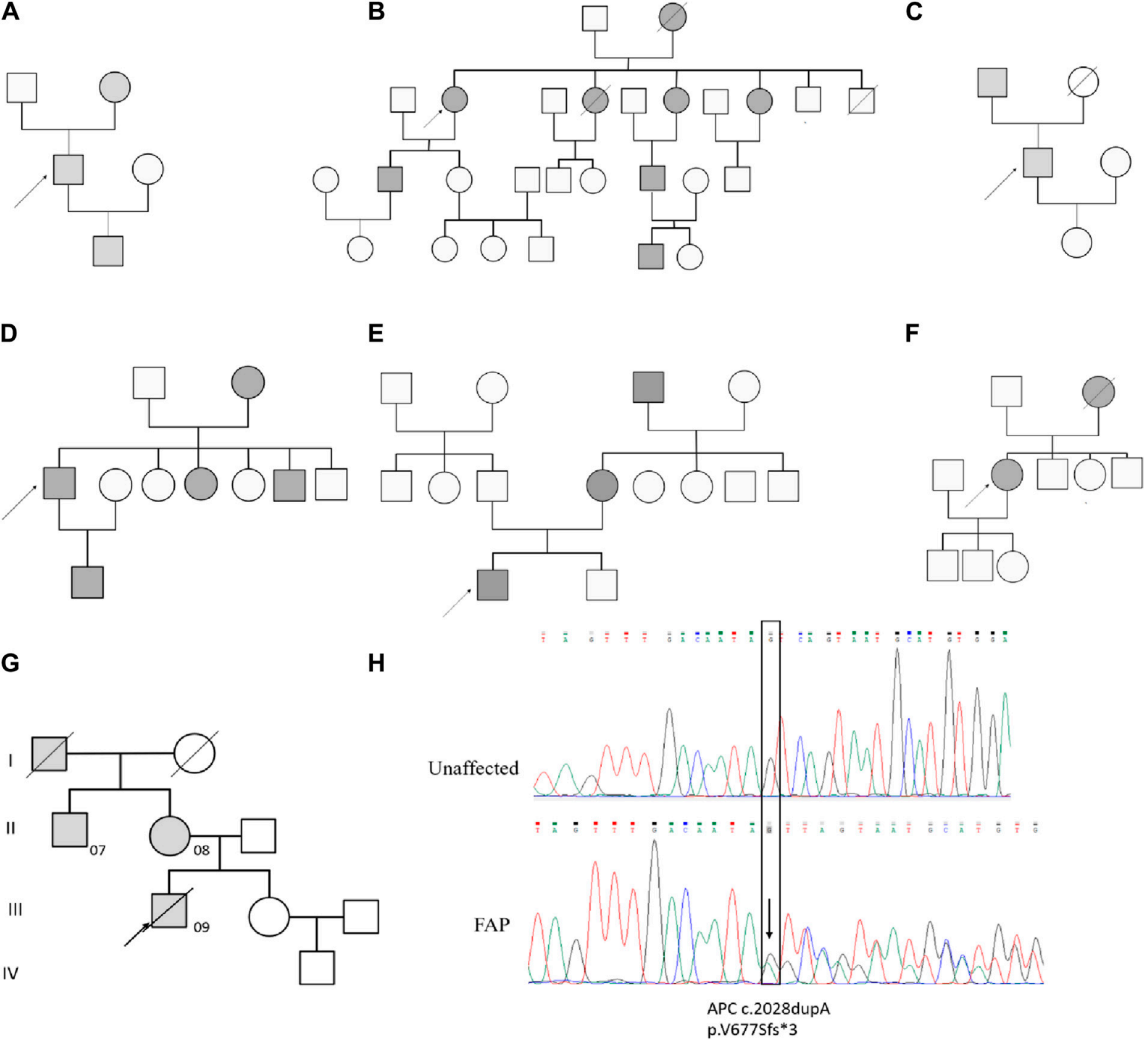
Pedigree analysis of FAP Patients. Pedigree analysis for FAP01 **(A)**, FAP02 **(B)**, FAP03 **(C)**, FAP04 **(D)**, FAP05 **(E)**, FAP06 **(F)** and FAP07-FAP09 **(G)**. The mutation of c.2028dupA (p.V677Sfs3) in blood sample of FAP.09 and an unaffected family member **(H)**.

The study cohort consisted of 9 patients diagnosed with FAP, with 3 patients (FAP07, FAP08, and FAP09) from the same family. A total of 36 samples were collected, including 9 peripheral blood samples, 9 tubular adenomas, 10 villous adenomas, and 8 carcinomas (Table 1). Among the patients, inherited germline mutation sites were identified in 8 out of 9 individuals. These mutations include FAP01 p.S1281* (COSM19212), FAP03 p.S384Tfs*19, FAP04 p.E1538* (COSM6041693), FAP05 p.Q1062* (COSM3696862), and FAP07-FAP09 p.V677Sfs*3. The pedigree analysis of FAP patients were shown in Figure 1.

**TABLE 1:** The germline mutation of *APC* in FAP cohort.

Patients	Age	Gender	Pathology number	Histological	Germline mutation (*APC*)	
					Gene mutation	Protein mutation
FAP01	33	Male	2015-10225A1	Cancer	c.3842C>G	p.S1281*(COSM19212)
			2015-10225A4	Villous		
			2015-10225C1	Tubular		
FAP02	52	Female	2016-04836A1	Cancer	NA	NA
			2016-04836C1	Villous		
			2016-04836C2	Tubular		
FAP03	51	Male	2016-06640A1	Villous	c.1150_1151ins19	p.S384Tfs*19
			2016-06640B1	Tubular		
FAP04	62	Male	2017-2279A1	Cancer	c.4612G>T	p.E1538*(COSM6041693)
			2017-2279B2	Villous		
			2017-2279B4	Tubular		
FAP05	25	Male	2018-05846A1	Villous	c.3184C>T	p.Q1062*(COSM3696862)
			2018-05846B1	Tubular		
FAP06	32	Female	2018-12236A1	Villous	NA	NA
			2018-12236B1	Tubular		
FAP07	54	Male	18R10492	Villous	c.2028dupA	p.V677Sfs*3
			18R10493	Tubular_1		
			18R10494	Tubular_2		
FAP08	52	Female	18R12363	Villous	c.2028dupA	p.V677Sfs*3
			18R12365	Tubular		
FAP09	38	Male	18R10488	Villous_1	c.2028dupA	p.V677Sfs*3
			19R03644	Villous_2		
			18R10487	Cancer_1		
			18R10489	Cancer_2		
			19R03641	Cancer_3		
			19R03642	Cancer_4		
			19R03643	Cancer_5		

### Novel germline mutation in pedigree analysis

A novel germline mutation, c.2028dupA (p.V677Sfs3), was identified in the family of FAP09 (Figure 1G, H). This mutation has not been previously reported in published literature or any existing databases. Genetic screening was conducted for p.V677Sfs3 on 5 individuals spanning four generations of this family. FAP09, the proband, had a grandfather (I1) who passed away from colon cancer at the age of 45, but it remains unclear if he carried the pathogenic mutation. The proband’s mother (FAP08,Ⅱ2) and uncle (FAP07, II1) were found to have the heterozygous p.V677Sfs3 mutation, and colonoscopy revealed multiple polyps in their cases. However, their sister (Ⅲ2) had a normal colonoscopy and did not carry the p.V677Sfs3 mutation. Similarly, their nephew (IV1) exhibited a normal phenotype and did not exhibit any genetic mutation. Hence, the p.V677Sfs3 mutation segregates independently from the phenotype in his family.

According to the guidelines established by the American College of Medical Genetics and Genomics (ACMG) (Sirohi et al., 2020), we have assessed the pathogenicity of the p.V677Sfs*3 variant as follows: The p.V677Sfs*3 variant is located in exon 14 of the *APC* gene, where an additional base has been inserted, leading to a frameshift mutation that results in premature termination of protein translation. This is considered a loss-of-function variant, meeting the criteria for strong evidence of pathogenicity (PVS1). Upon searching the 1,000 Genomes, gnomAD, and ESP databases, the p.V677Sfs* variant has not been identified in the general population, which aligns with the pathogenicity criterion PM2. The frequency of the variant is significantly higher in the affected population compared to the control population, which satisfies the pathogenicity criterion PS4. There is clear evidence of co-segregation of the mutation with the disease phenotype within families, which meets the pathogenicity criterion PP1. Based on the classification standards for pathogenic variants established by the ACMG, the *APC* p.V677Sfs*3 variant meets the pathogenicity criteria PVS1, PM2, PS4, and PP1, and is therefore classified as a pathogenic variant.

FAP09 underwent curative resection of colorectal cancer in 2016, but liver metastasis was detected in 2018. Unfortunately, FAP09 eventually succumbed to CRC recurrence 1 year after the discovery of liver metastasis. Since 2016, both FAP07 and FAP08 have been undergoing regular annual colonoscopic adenoma treatment. Tissue samples were collected from different historical stages of patients within this family to elucidate the evolutionary history of adenomas progressing to colorectal cancer in the FAP family.

### Somatic mutation landscape in the cohort

**FIGURE 2:**
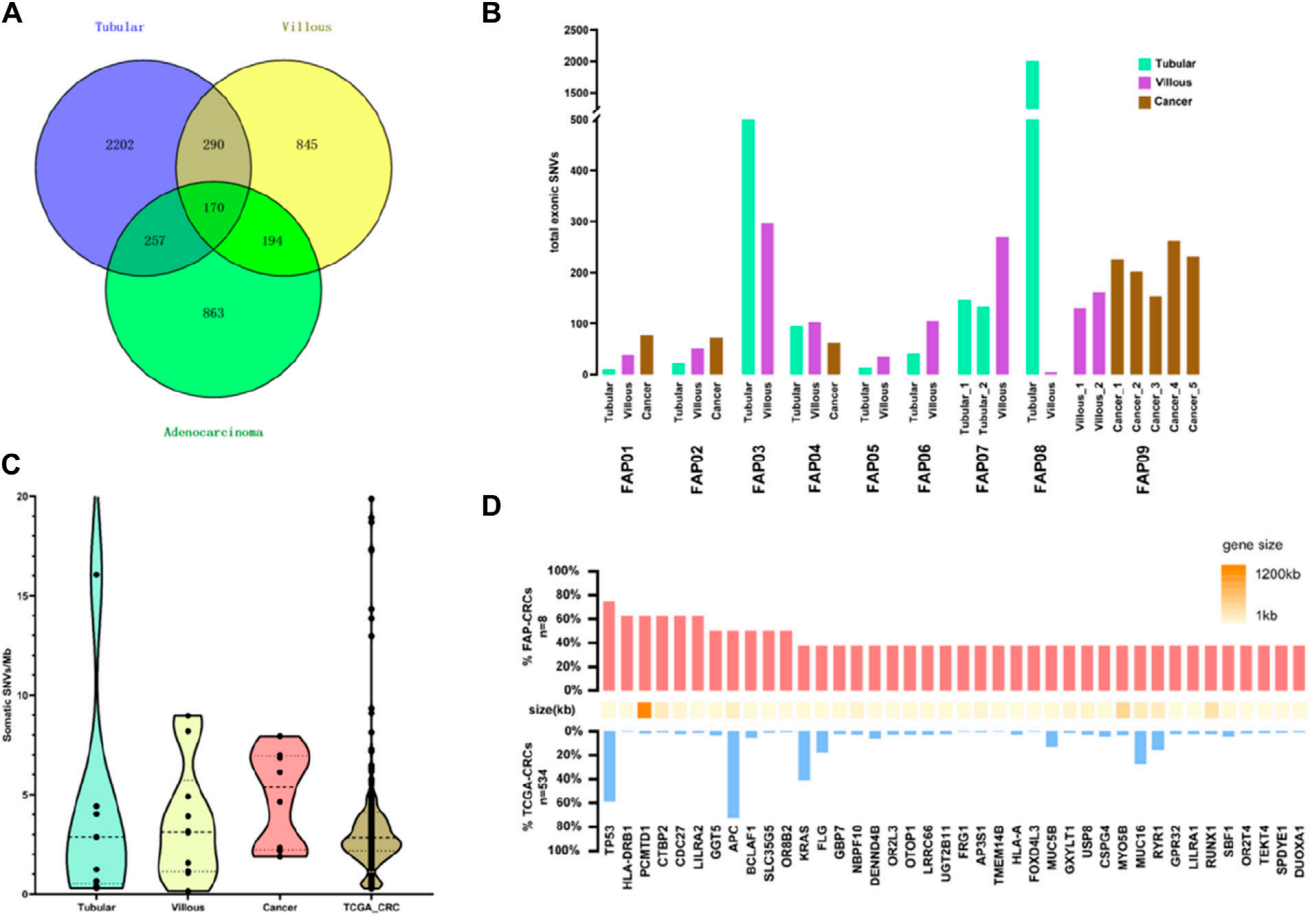
Analysis of single nucleotide alteration (SNA) burden in FAP. **(A)** Total SNVs in FAP-tubular, FAP-villus, FAP-CRC. **(B)** Total SNVs in adenomas and cancers in individual FAP patients. **(C)** Analysis of mutation burden in FAP-tubular, FAP-villus, FAP-CRC and S-CRC (data come from TCGA). **(D)** Analysis of recurrent mutation in FAP-CRC versus S-CRC (FAP-CRC n = 8, S-CRC n = 365, S-CRC data come from TCGA).

The somatic mutation landscape of the cohort was investigated (see somatic mutations list in Supplementary Data Sheet S2). Firstly, mutations present in the tissue specimens were subtracted from blood mutations. Next, these resulting mutations were further filtered based on their similarities to reproductive mutations in public databases and algorithms to isolate somatic mutations. A total of 1,484 somatic mutations were detected in eight carcinomas, 1,499 somatic mutations in 10 villous adenomas, and 2,919 somatic mutations in nine tubular adenomas (Figure 2A). It is worth noting that there were few common mutations shared between any two tissue samples within the same patient, indicating that the majority of somatic variants are random mutations (Supplementary Figure S1). Figure 2B illustrates the total number of exonic somatic variants (SVNs) in individual tissue samples. The mean quantities of SVNs were 108.0 (27, 206.5) in tubular adenomas, 122.0 (51.5, 309.8) in villous adenomas, and 290.5 (89, 388.5) in FAP-CRC. There is a tendency that the quantities of SVNs increased from tubular, villous to FAP-CRC. However, due to the limited sample size within each group and the substantial variability observed, traditional analysis of variance (ANOVA) did not reveal statistically significant differences (*p* = 0.50). To avoid bias in further analysis, tubular adenomas in FAP3 and FAP08, which had abnormally large numbers of SNVs, were excluded. The mutation burdens (MBs), measured as the number of mutations per mega-base, were analyzed for FAP-CRC and adenomas. FAP-CRCs exhibited a median mutation burden of 5.8 single nucleotide alterations (SNAs) per Mb, which was roughly twice as high as S-CRC (TCGA-CRCs, 2.5 SNAs/Mb). Additionally, both tubular adenomas and villous adenomas displayed considerable mutation burdens, with medians of 2.8 and 3.1 SNAs, respectively, suggesting the accumulation of somatic mutations from the early stages of cancer transformation (Figure 2C). Although statistical significance was not achieved due to the limited sample size, FAP-CRCs showed a relatively higher occurrence of TP53 mutations compared to S-CRC (Figure 2D).

### Recurrently mutated genes in FAPs cohort

**FIGURE 3:**
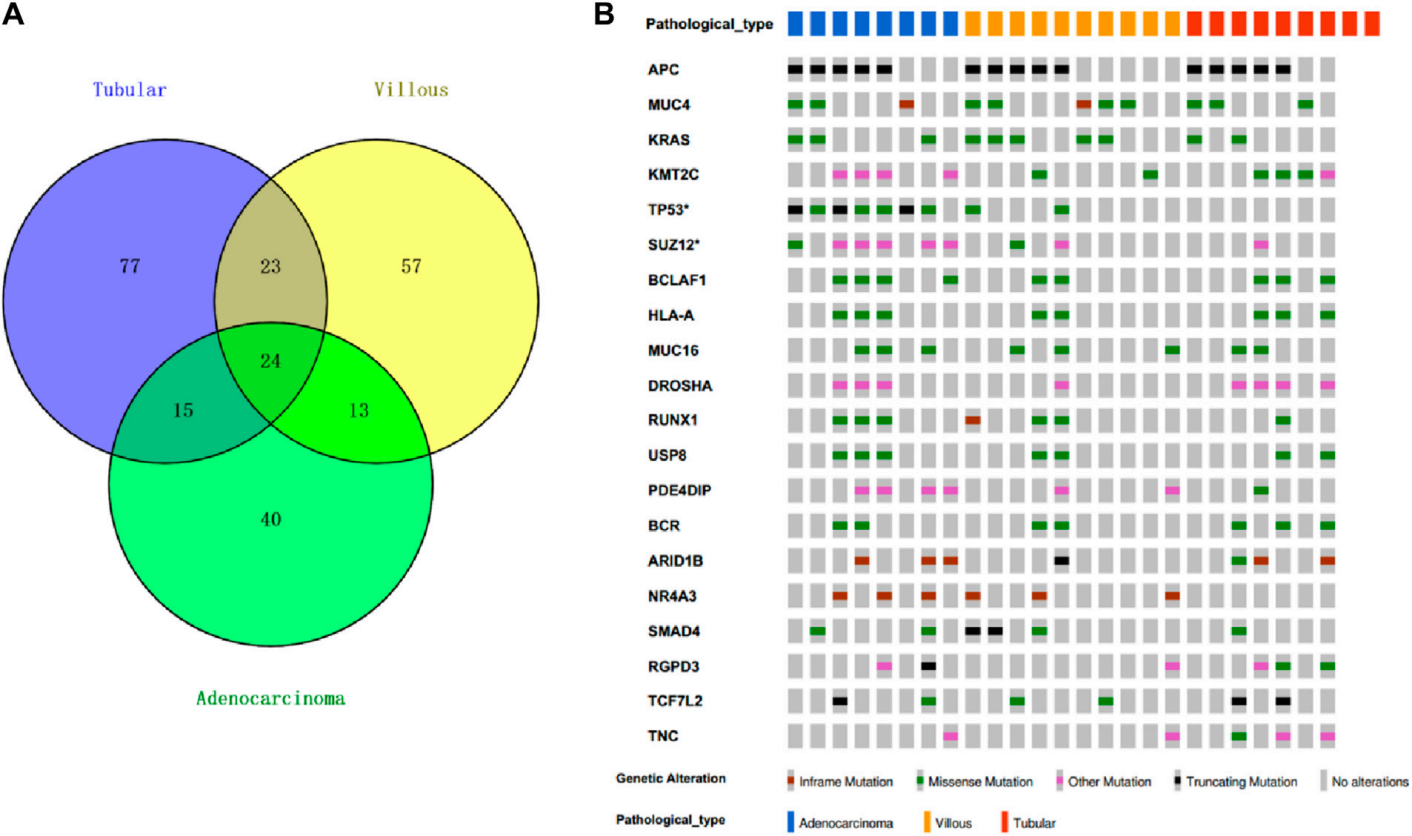
Top20 recurrent mutation in FAP cohort. **(A)** Cosmic mutations in FAP-tubular, FAP-villus, FAP-CRC. **(B)** Recurrent mutation including APC, MUC4, KRAS, KMT2C, TP53, SUZ12, BCLAF1, HLA-A, MUC16, DROSHA, RUNX1, USP8, PDE4DIP, BCR, AR1D1B, NR4A3, SMAD4, RGPD3, TCF7L2 and TNC.

By examining the whole-exome sequencing (WES) data from villous, tubular, and adenocarcinoma samples and comparing them with the oncogenic and tumor suppressor genes listed in the COSMIC database, we identified 24 genes that were shared among these samples (Figure 3A). In addition to the conventional recurrently mutated genes observed in FAPs, such as *APC*, *RAS*, and *TP53*, we also discovered novel recurrently mutated genes in the homologous recombination repair pathway. These genes include *SUZ12, KMT2C, BCLAF1, DROSHA, RUNX1, SMAD4, RGPD3,* and *ARID1B*. The presence of mutations in these genes suggests a state of genome instability.

The frequency of *APC* mutations, including protein-truncating mutations, was found to be the highest among all the lesions from patients with FAP (15 out of 25; mutation frequency = 69%) (Figure 3B). The occurrence of the second hit of *APC* may occur in the early phases of carcinogenesis since there was no significant difference in *APC* mutation frequency observed among tubular adenomas, villous adenomas, and carcinomas. *KRAS* missense mutations were found in carcinomas (3 out of 8; mutation frequency = 37.5%), in villous adenomas (5 out of 10; mutation frequency = 50%) and in tubular adenomas (2 out of 9; mutation frequency = 22.2%). Interestingly, it seems that villous adenomas had a higher *KRAS* mutation rate than carcinomas, although this was not statistically significant due to the limited sample size. Nearly all FAP-CRC samples (7 out of 8; mutation frequency = 87.5%) showed *TP53* mutations, including three truncating mutations and four missense mutations. Two villous adenomas with TP53 mutations were found to be complicated with regional canceration, indicating that *TP53* mutations occurred during the malignant transformation stage.

### Recurrently mutated genes in FAP09 family

**FIGURE 4:**
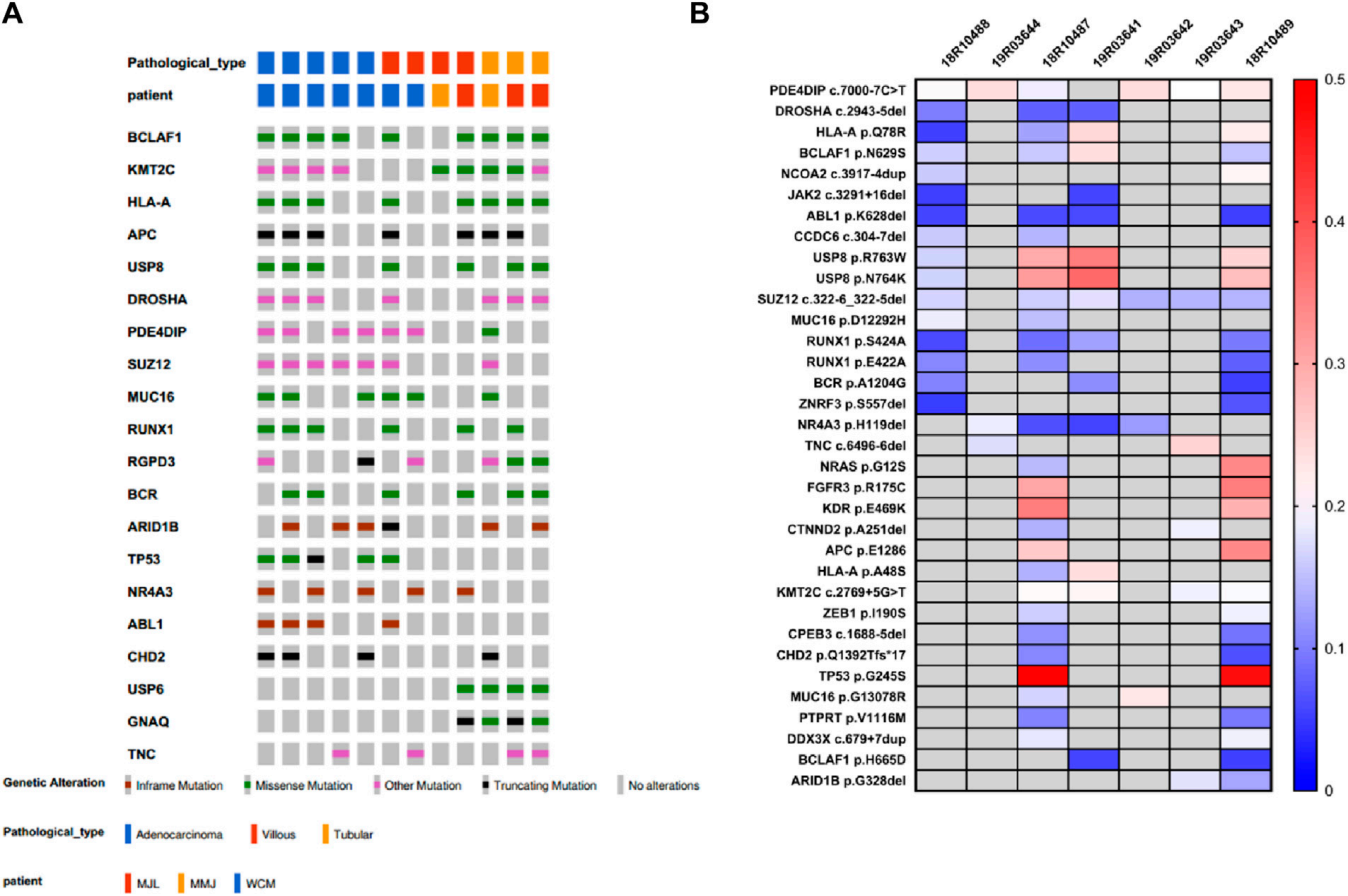
Top20 recurrent mutation in FAP.09 family **(A)** and FAP.09 patient **(B)**.

FAP07, FAP08, and FAP09 belong to the same family and carry the pathogenic variation p.V677Sfs*3. Within this family, the second hit of *APC* gene was detected in 3 out of 4 villous adenomas, 2 out of 3 tubular adenomas, and 3 out of 5 carcinomas (Table 2; Figure 4A). Interestingly, a specific somatic mutation, *APC* p.R1450*, was found in certain adenomas of all three patients. Mutation analysis revealed that *RAS* mutations, including *KRAS* and *NRAS,* were only detected in the tissues of carcinomas. Notably, the presence of the same mutation panel, with *NRAS* p.G12S and *TP53* p.G245S, was identified in 18R10487 (carcinoma from curative resection of colorectal cancer) and 18R10489 (carcinoma from liver metastases) (Figure 4B). Except for divergent mutations in *NRAS* and *TP53*, the two samples shared numerous somatic mutations, indicating a common origin.

**TABLE 2:** The recurrent mutation and mutation burden in FAP.09 family.

Patients	Pathology number	Histological types	*APC*	*KRAS*	*TP53*	*NRAS*	HRD-score	TMB	MSI
FAP07	18R10492	Villous	p.R1450*	NA	NA	NA	18	9.67	MSS
	18R10493	Tubular-1	NA	NA	NA	NA	8	6.17	MSS
	18R10494	Tubular-2	p.Q1429*	NA	NA	NA	5	6.53	MSS
FAP08	18R12363	Villous	p.R405*	NA	NA	NA	12	5.13	MSS
	18R12365	Tubular	p.R1450*	NA	NA	NA	15	6.28	MSS
FAP09	18R10487	CRC-cancer, primary	p.E1286*	NA	p.G245S	p.G12S	42	10.54	MSS
	19R03641	CRC-cancer, primary	p.H1490Ifs*17	NA	p.R196*	NA	21	7.70	MSS
	19R03642	CRC-cancer, primary	NA	p.G12V	p.R248W	NA	22	11.75	MSS
	19R03643	CRC-cancer, primary	NA	NA	NA	NA	21	9.49	MSS
	19R03644	Villous	NA	NA	NA	NA	13	6.35	MSS
	18R10488	Villous	p.R1450*	NA	p.Y220C	NA	24	7.80	MSS
	18R10489	CRC-cancer, metastasis	p.E1286*	NA	p.G245S	p.G12S	47	10.06	MSS

In the mutation burden analysis, the mutation burden for tubular adenomas was calculated as 6.28 (6.17, 6.53), for villous adenomas it was 10.06 (5.13, 4.54), and for carcinomas it was 10.07 7.7, 11.75). There are significant difference among them (*p* = 0.01) in one way ANOVA analysis. In the analysis of homologous recombination deficiency (HRD), the HRD score for tubular adenomas was 6.28 (ranging from 5.0 to 15.0), for villous adenomas it was 15.50 (ranging from 12.0 to 24), and for carcinomas it was 22.0 (ranging from 21.0 to 45.0). There is a significant difference between tubular adenoma and cancer with *p* = 0.03 by *t*-test. Although the statistical difference between tubular adenoma and villous adenoma is not significant, it may be due to the limited number of specimens. There is a clear tendency of progressively increasing mutation burden and HRD score from tubular adenomas to villous adenomas and ultimately to carcinomas, indicating a state of genome instability.

### Evolutionary history of SNAs

**FIGURE 5:**
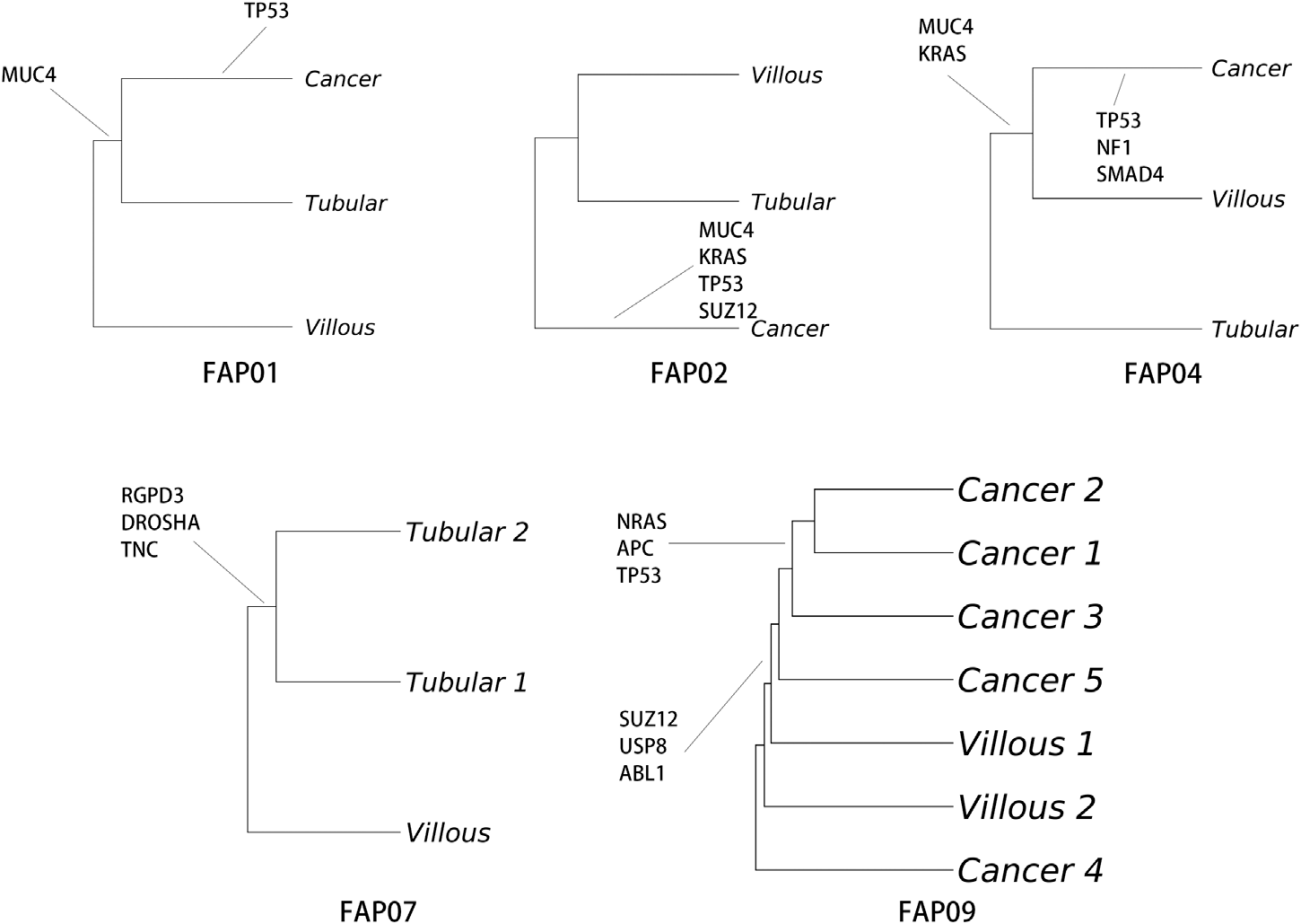
Analysis of single nucleotide alteration (SNA) phylogeny in FAP-CRC. Phylogenetic trees were produced using maximum parsimony and multiregional whole-exome sequencing (WES). Branches are labelled with SNA drivers.

To understand the development and evolution from adenomas to carcinomas in FAP, phylogenetic trees of tissue samples from various sites were assessed. The *TP53* mutation was identified as the primary driver gene for malignant transformation, except in the case of FAP.07. Remarkably, the conventional driver mutations (*TP53, RAS,* and *SMAD4*) consistently emerged simultaneously rather than being acquired sequentially after the initiation of cancer growth, as exemplified in FAP.02, FAP.04, and FAP.09 (Figure 5). Based on the current data, a linear evolution relationship from tubular adenomas to villous adenomas and ultimately to carcinomas cannot be determined. The malignant transformation from adenomas to carcinomas appears to occur abruptly, accompanied by mutations in driver genes.

**FIGURE 6:**
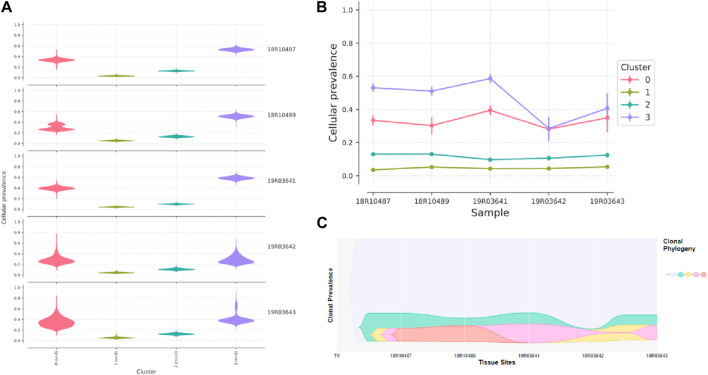
Graphical representation of clonal evolution from the primary tumor to metastasis in patient of FAP.09, with cellular prevalence in density **(A)**, cellular prevalence in parallel-coordinates **(B)** and clonal prevalence (C). The founding clone in the primary tumor, contained somatic mutations in MUC2, MUC5B, KMT2C, and SUZ12, all of which are recurrent in CRC cancer and probably relevant for pathogenesis. Subsequently, one subclone within the founding clone evolved to become the dominant clone at metastasis by acquiring additional mutations, including recurrent mutations in NRAS p.G12S and TP53 p.G245S mutation.

In the case of FAP.09, depth sequencing was performed on five carcinomas, allowing for accurate quantification of mutant allele frequencies and estimation of tumor clonal population sizes. Through the analysis of mutation clustering, four distinct clones with different sets of mutations were identified in the carcinomas of patient FAP.09 (Figure 6). The sample labeled as 19R03643 was presumed to be the initial clone, with median mutant allele frequencies of 34.98%, 5.38%, 12.42%, and 40.65% observed for clusters 0 to 3, respectively. Clone 0 serves as the “founding” clone, from which the other subclones are derived, and is presumed to be present in nearly all tumor cells. Clones 1, 2, and 3 are believed to have evolved from clone 0. It is likely that a single cell from clone three acquired a specific set of mutations, giving rise to clone 4, which subsequently became the dominant clone possessing metastatic capabilities. The *NRAS p.G12S* and *TP53 p.G245S* mutations are the most probable candidate genes in clone 4, providing a significant selective advantage for proliferation and metastasis.

## Discussion

Familial adenomatous polyposis (FAP) is an autosomal dominant colorectal cancer predisposition syndrome, typically characterized by the presence of numerous (ranging from hundreds to thousands) polyps in the epithelium of the large intestines caused by germ line mutations in the APC gene (Aelvoet et al., 2022). We reconstructed the evolutionary history of FAP-CRC by employing multi-region Whole-Exome Sequencing (WES) analysis, focusing on a specific FAP family. By comparing the mutation signatures of tubular adenomas, villous adenomas and FAP-CRC, we found that adenomas exhibited lower mutational rates than FAP-CRC and recurrent alterations were observed in well-known chromosomal instability (CIN) genes (*APC, RAS, SMAD4* and *TP53*) and DNA damage repair genes (*SUZ12, KMT2C, BCLAF1, RUNX1,* and *ARID1B*) in the adenomas, suggesting the presence of genomic instability. Furthermore, a progressive increase in the HRD score (a measure of “genomic scars”) was observed from tubular adenomas to villous adenomas and ultimately to carcinomas. *TP53* was identified as the primary driver gene for adenoma-carcinoma transition, and the driver mutations consistently emerged simultaneously rather than being acquired sequentially from adenomas to carcinomas. Precancerous polyps in FAP already exhibit considerable mutation burden, as reported in previous studies. Li et al., found that pathogenic events may occur long before the appearance of clinically identifiable adenomas, even in a macroscopically normal epithelium in FAP (Li et al., 2020). Borras et al., reported that at least 23% of somatic mutations are present in at-risk mucosa prior to adenoma initiation (Borras et al., 2016).

The occurrence of a second hit within the *APC* gene, predominantly protein-truncating mutations, remains the most frequently observed recurrent event observed in our FAP cohort, with a mutation frequency of 69%. The position and type of this secondary hit in FAP polyps are influenced by the specific localization of the underlying germline mutation (Crabtree et al., 2003). A ‘just-right’ signaling model has been proposed regarding the second hit in the *APC* gene, suggesting that somatic mutations in APC are selected based on precise activation levels of beta-catenin signaling (Albuquerque et al., 2002). It was interesting that we identified the somatic mutation *APC* p.R1450* present in the villous adenoma of FAP07, tubular adenoma of FAP08, and villous adenoma of FAP09. It is plausible that this mutation site was selected based on the ‘just-right’ signaling model.

Previous studies have revealed the complexity of potential driver genes involved in adenoma-carcinoma transition in FAP. Alberici et al. found that few adenomas have the same set of mutated driver genes, apart from *APC, KRAS* and *WTX* mutations (Rashid et al., 2016). While they observed a high prevalence of *WTX* mutation, unfortunately it was not recurrent in other studies. Li et al., identified potential driver genes, including *APC, KRAS* and *TP53*, although the mutation frequency of *TP53* (8%) was relatively low (Li et al., 2020). In a relatively large FAP study cohort comprising 35 FAP patients from different families, the somatic spectrum of FAP-CRC was found to be similar to the early-onset CRCs, with higher *TP53* (94.1%), lower somatic APC mutations (65.7%), and higher *KRAS* mutation rate (58.5%). In light of the high prevalent *TP53* mutations in FAP-CRC, the researchers suggested that *TP53* ctDNA could be a novel tool for optimizing the timing of surgery (Ge et al., 2022). In alignment with this study, we identified *TP53* as a major driver gene in the adenoma-carcinoma transition. Nearly all FAP-CRC samples (7/8; mutation frequency = 87.5%) harbored *TP53* mutations. Two villous adenomas with *TP53* mutations were observed to be associated with regional carcinogenesis, indicating that *TP53* mutations occurred during the process of malignant transformation.

CRC shows variable underlying molecular changes with two major mechanisms of genetic instability: chromosomal instability (CIN) and microsatellite instability (Muller et al., 2016). In the case of FAP-CRC, the majority of cases are microsatellite stable (MSS). Recurrent alterations found in our cohort including known chromosomal instability genes (*APC, RAS, SMAD4* and *TP53*) and DNA damage repair genes (*SUZ12, KMT2C, BCLAF1, RUNX1,* and *ARID1B*). Moreover, we observed a tendency of progressively increased HRD score from tubular adenomas to villous adenomas and ultimately to carcinomas, indicating a state of genomic instability. CIN is considered one of the major types of genomic instability observed in CRC. Although the function of *APC* initiates the adenoma-carcinoma transition in the majority of CRCs through constitutive activation of Wnt/beta-catenin signaling, the APC gene also represents a candidate chromosome instability (CIN) gene in CRC. Chromosome instability has been observed in colorectal tumor cells by a dominant mutation in APC (Green and Kaplan, 2003). In addition, colon cancer cells with *APC* mutations have weakened kinetochore-microtubule interactions (Tighe et al., 2004). Apart from *APC*, other genes including *RAS* (Berg and Soreide, 2012), *SMAD4* (Woodford-Richens et al., 2001) and *TP53* (Weiss et al., 2010) also participated in chromosomal instability, resulting in aneuploidy and promoting tumor progression.

Recurrent genes in DNA damage repair pathways, including *SUZ12, KMT2C, BCLAF1, RUNX1,* and *ARID1B. SUZ12* is a component of the NuA4 histone acetyltransferase complex, which plays a key role in chromatin remodeling and gene expression regulation. Knockdown of *SUZ12* using small interfering RNA (siRNA) has been shown to reduce p53 stability and DNA repair in hepatocellular carcinoma (Wang et al., 2011). *KMT2C* is a histone methyltransferase that participates in chromatin remodeling. Downregulation of *KMT2C* in bladder cancer cells leads to DNA damage and genomic instability (Rampias et al., 2019). *BCLAF1*, a functional partner of BACH1, participates in DNA damage repair and the maintenance of genomic stability (Jiang et al., 2022). The *RUNX* family has been identified as a novel multifaceted guardian of the genome (Dutta et al., 2023). Dysregulation of *RUNX1* genes can promote genomic instability in solid cancers by impairing DNA repair mechanisms (Krishnan, 2023). *ARID1B* is a key component of BAF complex of the SWI/SNF chromatin-remodeling family, which regulates gene expression during cellular development and influences the DNA damage response (Watanabe et al., 2014).

Clonal evolution showed that a set of driver mutations always consistently emerged simultaneously rather than being acquired sequentially from adenomas to carcinomas, which suggested CRCs evolved according to the “Big Bang” model proposed for S-CRC evolution (Sottoriva et al., 2015).

In summary, our study provides a comprehensive understanding of the genomic landscapes transitioning from adenoma to carcinoma. The carcinogenesis process in FAP-CRC supports the classical cancerization model, where *APC* mutations lead to the activation of the WNT signaling pathway and chromosomal instability (CIN). This, in turn, cause mutations in other putative CIN genes (e.g., DNA repair, chromatin remodeling), ultimately resulting in the development of microsatellite stable (MSS) tumors. FAP-CRC has an elevated HRD score, suggesting potential sensitivity to poly (ADP-ribose) polymerase (PARP) inhibitors and platinum-based therapies.

## Data Availability

The data presented in the study are deposited in the CNSA database (https://db.cngb.org/cnsa), with accession number CNP0005680.
